# Statistical analysis of Covid-19 mortality rate via probability distributions

**DOI:** 10.1371/journal.pone.0274133

**Published:** 2022-10-27

**Authors:** Muhammad Farooq, Muhammad Ijaz, Muhammad Atif, Tahani Abushal, Mahmoud El-Morshedy

**Affiliations:** 1 Department of Statistics, University of Peshawar, Peshawar, Khyber Pakhtunkhwa, Pakistan; 2 Department of Mathematics and Statistics, The University of Haripur, Haripur, Khyber Pakhtunkhwa, Pakistan; 3 Department of Mathematical Sciences, Umm Al-Qura University, Makkah Al Mukarramah, Saudi Arabia; 4 Department of Mathematics, College of Science and Humanities in Al-Kharj, Prince Sattam Bin Abdulaziz University, Al-Kharj, Saudi Arabia; Universita degli Studi di Milano, ITALY

## Abstract

Among other diseases, Covid 19 creates a critical situation around the world. Five layers have been recorded so far, resulting in the loss of millions of lives in different countries. The virus was thought to be contagious, so the government initially severely forced citizens to keep a distance from each other. Since then, several vaccines have been developed that play an important role in controlling mortality. In the case of Covid-19 mortality, the government should be forced to take significant steps in the form of lockdown, keeping you away or forcing citizens to vaccinate. In this paper, modeling of Covid-19 death rates is discussed via probability distributions. To delineate the performance of the best fitted model, the mortality rate of Pakistan and Afghanistan is considered. Numerical results conclude that the NFW model can be used to predict the mortality rate for Covid-19 patients more accurately than other probability models.

## Introduction

There are various daily life examples where the failure rate has a wide range of values. For example, the failure rate of a healthy person is constant over some specified time and hence follows an exponential distribution. Similarly, the failure rate at the time of surgery is maximal but over time it declines and therefore follows a descending Weibull distribution, but if a person fails to respond to treatment and their condition worsens over time, such a scenario is predicted. by reducing the Weibull distribution [1]. There are cases where a person’s condition worsens in the early stages and then begins to recover after a specific time, for example, the failure rate of Covid19 patients is highest in the first two weeks, then decreases after two weeks, and is therefore unpredictable by both the exponential distribution and the Weibull distribution. Therefore, these models cannot be used to deal with such non-monotonic failure rate functions. The coronavirus pandemic has shocked the world and created anxiety among human beings, which has compelled researchers to predict the situation as it varies from country to country. These forecasts allow government agencies to respond to any unforeseen circumstances and plan accordingly. More interestingly, many probabilistic models have been used to predict patterns of an uncertain future event, for example, Mdzinarishvili and Sherman [2] tested a Weibull-like model that cancer risk will increase with age, but these risks will turn around. Polymenis [3] predicted intertemporal mortality for Covid patients by applying an exponential distribution. Zuo et al. [4] modelled the total number of Covid19 deaths using a new flexible extended Weibull model. Al-Jibory and El-Zaart [5] used the Weibull distribution to create masks for Alzheimer’s patients, Huysmans et al [6] evaluated the initial fracture load of the premolars directly after restoration and reinforcement using the Weibull distribution. Vergu et al. [7] point out that most pandemic situations are based primarily on exponential distributions, but the situation is not always the same, which can change the dynamics. of the pandemic. Under such circumstances, to model the mortality rate of Covid-19 patients accurately, a new probability model can be created to help the government to arrange hospital facilities like hospital beds, isolation rooms, etc. For other research studies conducted on Covid-19, we refer to [8, 9].

Researchers have been very interested in designing a new probabilistic model for modelling complex data, especially in reliability engineering and survival rate analysis. In this paper, the main objective of the paper is to choose the best fitted model for covid-19 and then discuss its structural properties and a simulation study. The best fitted model is New Flexible Weibull (NFW).

## Methodology

Multiple distribution families were introduced by researchers to overcome the problem of existing distribution families. For example, Ijaz et al. [10] introduced the family of Gull Alpha power Weibull distributions that are not only flexible, but can also deal with non-monotonic and non-monotonic hazard rate functions [8]. A similar study was performed by Farooq et al. [11] to introduce a family of distributions called the Flexible Exponential Family (FEF) of distributions. The Weibull [10] distribution is taken as the base distribution. The proposed distribution has been applied to the “Total deaths per million in Pakistan”, which shows that the proposed distribution works better and is more predictable than the other distributions. To deal with outliers in a data set, Farooq et al. developed a distribution generator called New Flexible Family (NFF). They are suggested to show better performance than other lifetime distributions while modelling lifetime data in the presence of extreme values. Alzaatreh et al. [12] developed the Weibull-X distribution which provides more flexibility in lifetime data while Cordeiro et al. [13] worked on the type-I half-logistic family of distributions. For a detailed discussion of distribution families, we refer to [14–24].

The research work done in the paper can be summarized as follows, first we discuss the best fitted model and its special cases. Some mathematical properties have been derived with their numerical results in the next section. The real significance is supported by using the two data sets and then concluding the paper.

A random variable *X* is said to be a New Flexible Family (NFF) of distributions if it holds the following cumulative distribution function (CDF) and probability density function (PDF)

$$F\left(x\right)=\frac{F\left(y\right)e^{F\left(y\right)}}{e}$$ (1)

$$f\left(x\right)=\frac{f\left(y\right)e^{F\left(y\right)}+f^{2}\left(y\right)e^{F\left(y\right)}}{e}$$ (2)

where “e” is the exponential function and *F*(*y*) is the CDF of the baseline distribution.

### Special form of the NFF

In this section, the special form of NFF is derived by using the CDF of the Weibull distribution called the NFW distribution. The CDF of the Weibull distribution [15] is presented by

$$F\left(x\right)=1-e^{-ax^{b}},\;x>0$$ (3)

where a and b represent the scale and shape parameters, respectively.

Using (3) in (1), the CDF and PDF of NFW are

$$F\left(x\right)=\frac{\left(1-e^{-ax^{b}}\right)e^{1-e^{-ax^{b}}}}{e}\;a,b>0$$ (4)

$$f\left(x\right)=abx^{b-1}\left(2e^{ax^{b}}-1\right)e^{-e^{-ax^{b}}-2ax^{b}}$$ (5)

**Fig 1:**
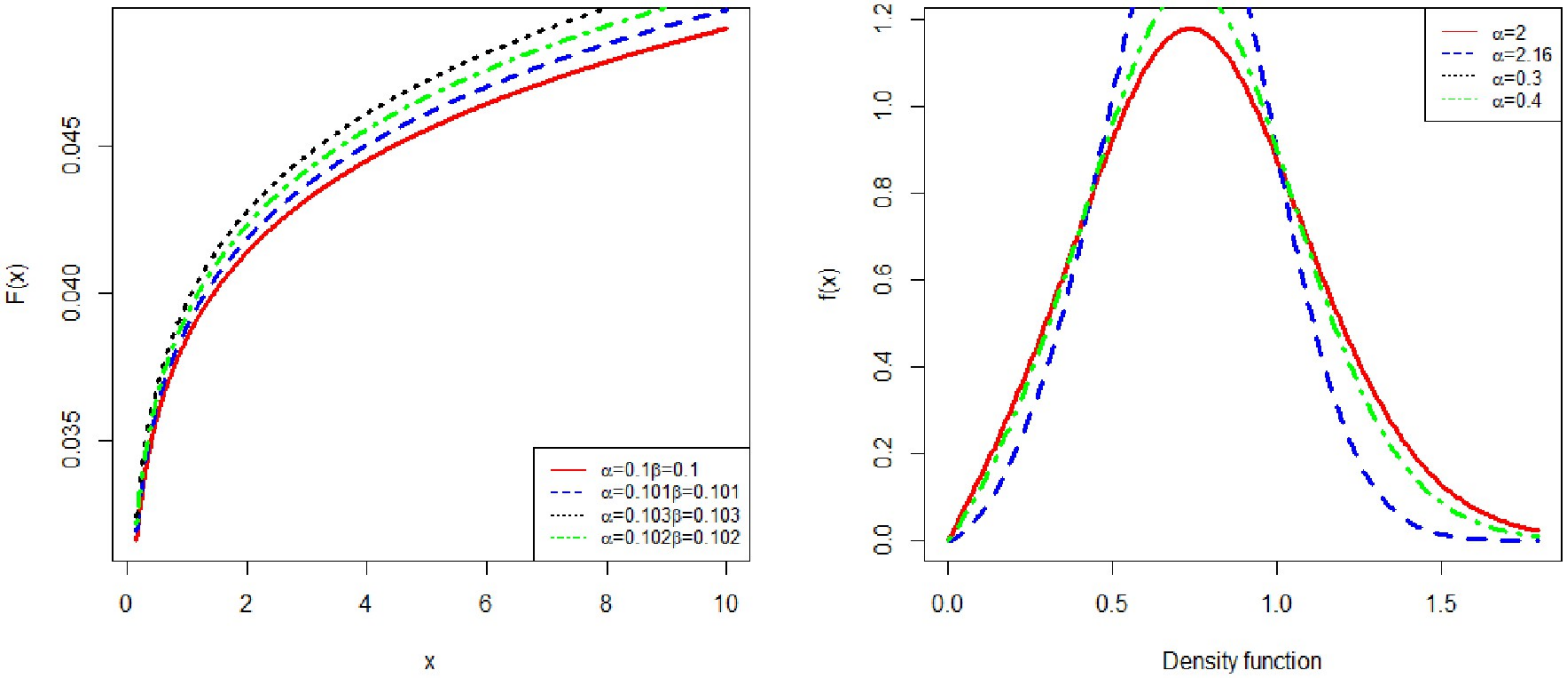
Plots of the CDF and PDF of NFW.

Fig 1 presents various shapes of the CDF and PDF with a different set of parameter values.

#### Statistical properties

In this section, various statistical properties of the proposed lifetime model have been discussed and is given below.

*The survival and hazard rate function*. The survival and hazard rate functions are respectively defined by

$$S\left(x\right)=e^{-ax^{b}-e^{-ax^{b}}}$$ (6)

$$h\left(x\right)=abx^{b-1}\left(2e^{ax^{b}}-1\right)e^{-ax^{b}}$$ (7)

**Fig 2:**
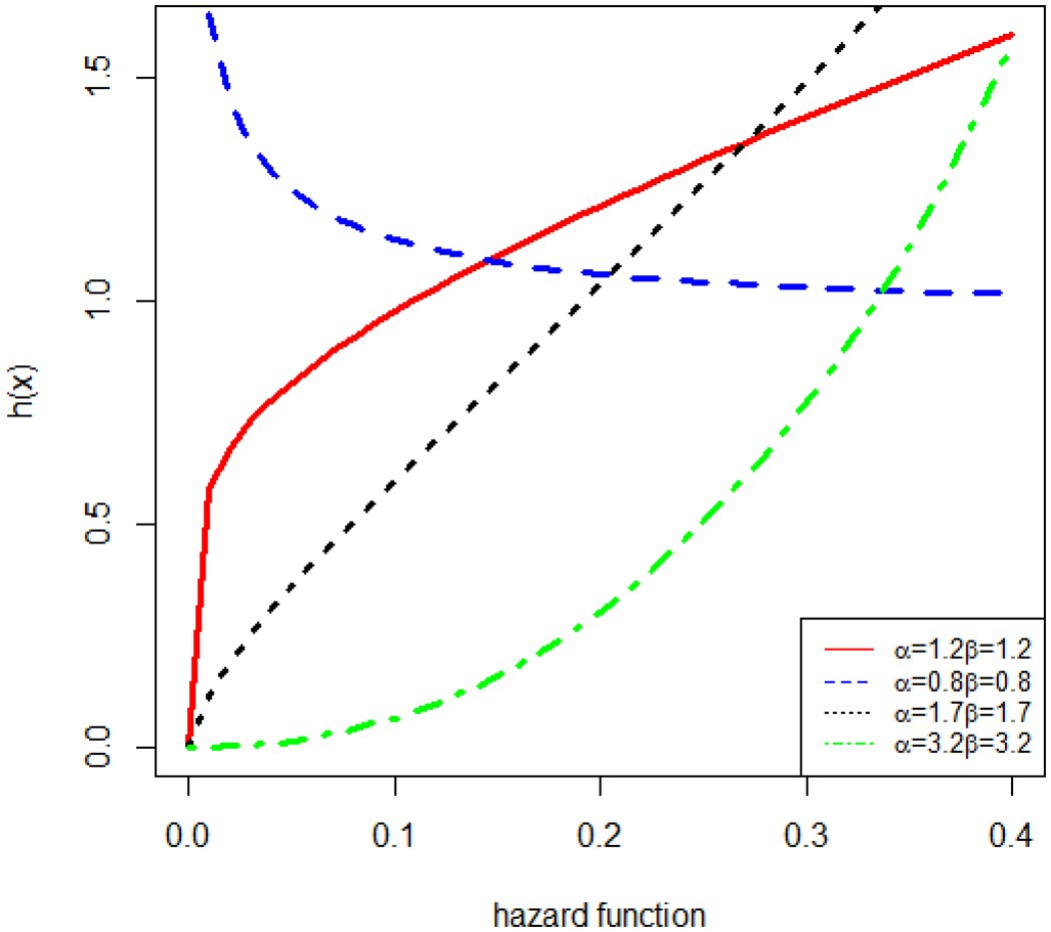
Plots of the hazard rate function of NFW.

Different forms of the hazard ratio function with different parameter values are identified in Fig 2. The graph shows that it can track rising, falling, J-shaped, and inverted J-shaped hazard rates.

*Quantile function*. The quantile function depends on different levels of *q* and can be defined by using the following relation

$$p\left(X\leq x\right)=q$$

Using (4) and simplify the result, we get

$$x=\sqrt[{b}]{\frac{\text{log}\left(\frac{1}{1-W\left(e*q\right)}\right)}{a}}$$ (8)

where “e” is the exponent, q is the quantile function and “W” is a Lambert function. Table 1 defines the numerical results of the expression given in (8).

**Table 1:** Numerical results with different values of parameter and quantiles.

a	b	q	Numerical results (x)
0.1	20	0.25	1.126619
0.1	0.5	0.50	211.2654
1	0.1	0.75	1764.539
1	1	0.25	1.085287
10	1	0.50	0.1453497
10	10	0.75	0.8559853
2	0.2	0.50	0.2027314
2	0.5	0.75	1.114978

*Order statistics*. Let *X*_*i*_ (*i* ≤ *n*) be an ordered stochastic variable then the *i*^*th*^ order statistic PDF is given by

$$f\left(i,n\right)\left(x\right)=\frac{n!}{\left(i-1\right)!\left(n-i\right)!}f\left(x\right)F{\left(x\right)}^{i-1}{\left(1-F\left(x\right)\right)}^{n-i}$$ (9)

Using Eqs (3) and (4), the smallest and largest order statistics of NFW can be obtained respectively by using *i* = 1 and *i* = *n* as

$$f_{\left(1,n\right)}\left(x\right)=nabx^{b-1}\left(2e^{ax^{b}}-1\right)e^{-e^{-ax^{b}}-2ax^{b}}{\left(1-\left(1-e^{-ax^{b}}\right)e^{-e^{-ax^{b}}}\right)}^{n-1}$$ (10)

$$f_{\left(n,n\right)}\left(x\right)=nabx^{b-1}\left(2e^{ax^{b}}-1\right)e^{-ne^{-ax^{b}}-2ax^{b}}{\left(1-e^{-ax^{b}}\right)}^{n-1}$$ (11)

*Skewness and kurtosis*. The quantile function is used to measure the effect of the shape parameters on skewness and kurtosis. Henceforth, using the quantile function of the NFW distribution, we have obtained the expressions for skewness and kurtosis with the following forms

$$S_{B}=\frac{Q\left(\frac{6}{8}\right)+Q\left(\frac{2}{8}\right)-2Q\left(\frac{4}{8}\right)}{Q\left(\frac{6}{8}\right)-Q\left(\frac{2}{8}\right)}$$ (12)

$$K_{M}=\frac{Q\left(\frac{7}{8}\right)+Q\left(\frac{3}{8}\right)-Q\left(\frac{5}{8}\right)-Q\left(\frac{1}{8}\right)}{Q\left(\frac{6}{8}\right)-Q\left(\frac{2}{8}\right)}$$ (13)

where *Q* describe different quartile values.

Table 2 describes some numerical results for the skewness and kurtosis for different parameter values.

**Table 2:** Skewness and kurtosis for different values of parameter.

a	b	Skewness	Kurtosis
0.1	20	0.1305604	1.139097
0.1	0.5	0.4303544	1.663804
1	0.1	0.954809	16.0037
1	1	0.2826349	1.327727
10	1	0.2826349	1.327727
10	10	0.1387393	1.146186
0.01	0.01	1	1.176981e+12
2	0.2	0.7539918	3.843164
2	0.5	0.4303544	1.663804
0.2	5	0.1550559	1.161267

*Rth moments*. The rth moment of the proposed model can be obtained as

$$E(x^{r})=\int_{0}^{\infty }x^{r}f\left(x\right)dx$$

Using Eq (5), we get

$$E(x^{r})=\int_{0}^{\infty }x^{r}abx^{b-1}\left(2e^{ax^{b}}-1\right)e^{-e^{-ax^{b}}-2ax^{b}}$$

$$E(x^{r})=2\int_{0}^{\infty }abx^{\text{r}(b-1)}e^{-e^{-ax^{b}}-ax^{b}}dx-\int_{0}^{\infty }abx^{\text{r}(b-1)}e^{-e^{-ax^{b}}-2ax^{b}}dx$$ (14)

Finally, we derived the following result

E(xr)=2a∑k=0∞−akk*k!r(b−1)+2bk−akr(b−1)+2bk−a∑k=0∞2k−akk*k!r(b−1)+2bk−akr(b−1)+2bk (15)

To obtain the first four moments, we have to put r = 1,2,3, and 4 in (15). The expression for each is as under

E(x)=2a∑k=0∞−akk*k!b+1bk−akb+1bk−a∑k=0∞2k−akk*k!b+1bk−akb+1bk (16)

E(x2)=2a∑k=0∞−akk*k!2bbk−ak2bbk−a∑k=0∞2k−akk*k!2bbk−ak2bbk (17)

E(x3)=2a∑k=0∞−akk*k!3b−1bk−ak3b−1bk−a∑k=0∞2k−akk*k!3b−1bk−ak3b−1bk (18)

E(x4)=2a∑k=0∞−akk*k!4b−2bk−ak4b−2bk−a∑k=0∞2k−akk*k!4b−2bk−ak4b−2bk (19)

## Applications

In this section, data on Covid-19 death rates in Pakistan and Afghanistan were reviewed to describe its practical applications. The dataset taken from Coronavirus (Covid19) Pandemic Statistics and Research (https://github.com/owid/covid19data) includes daily death rates per million people in Pakistan from May 2, 2020, until July 4, 2021.

The performance of the proposed model is assessed by the following goodness of fit measures

$$A=-n-\frac{1}{n}\sum_{i=1}^{n}\left(2i-1\right)\left[\text{log}\;F\left(X_{i}\right)+\text{log}\left(1-F\left(X_{n-i+1}\right)\right)\right]$$

$$W={\sum_{i=1}^{n}\left[F\left(X_{i}\right)-\frac{2i-1}{2n}\right]}^{2}+\frac{1}{12n}$$

$$AIC=-2L+2p,\;AICc=AIC+\frac{2p(p+1)}{n-p-1},\;CAIC=-2L+P\left\{\text{log}(n)+1\right\}$$

$$BIC=P\;\text{log}\left(n\right)-2L,\;HQIC=-2L+2P\;\text{log}\left\{\text{log}(n)\right\}.$$

where, $L=L(\hat{\psi };y_{i})$ is the maximized likelihood function and *y*_*i*_ is the given random sample, $\hat{\psi }$ is the maximum likelihood estimator and *p* is the number of parameters in the model.

It should be noted that the model with the fever value of these criteria is considered the best model among others. For more detailed information about the goodness of fit measures, we refer to [25–28].

### Data set 1: Covid-19 data for Pakistan (Total death rate in Millions)

0.009, 0.014, 0.014, 0.023, 0.027, 0.032, 0.036, 0.041, 0.05, 0.054, 0.063, 0.095, 0.118, 0.122, 0.154, 0.181, 0.186, 0.213, 0.24, 0.258, 0.276, 0.294, 0.299, 0.389, 0.412, 0.421, 0.435, 0.503, 0.579, 0.611, 0.647, 0.761, 0.797, 0.91, 0.96, 1.073, 1.145, 1.218, 1.272, 1.322, 1.412, 1.553, 1.743, 1.888, 1.992, 2.069, 2.155, 2.327, 2.553, 2.648, 2.712, 2.879, 2.983, 3.196, 3.336, 3.445, 3.486, 3.776, 3.776, 3.952, 4.088, 4.251, 4.459, 4.604, 4.83, 4.984, 5.129, 5.283, 5.419, 5.546, 5.704, 5.962, 6.315, 6.714, 6.985, 7.338, 7.642, 8.013, 8.321, 8.76, 9.063, 9.358, 9.833, 10.209, 10.666, 11.15, 11.15, 11.549, 12.354, 12.852, 13.468, 14.002, 14.618, 15.311, 15.849, 16.252, 16.728, 16.999, 17.669, 17.936, 18.267, 18.643, 18.864, 19.485, 19.897, 20.25, 20.603, 20.603, 20.911, 21.558, 21.907, 22.282, 22.559, 22.898, 23.192, 23.527, 23.84, 24.084, 24.383, 24.564, 24.564, 24.999, 25.207, 25.347, 25.528, 25.7, 25.845, 26.09, 26.198, 26.357, 26.357, 26.447, 26.551, 26.674, 26.818, 26.941, 26.941, 27.054, 27.158, 27.158, 27.226, 27.321, 27.398, 27.47, 27.534, 27.602, 27.67, 27.747, 27.792, 27.855, 27.896, 27.955, 27.955, 28.023, 28.073, 28.109, 28.154, 28.208, 28.267, 28.267, 28.317, 28.371, 28.403, 28.444, 28.448, 28.466, 28.494, 28.512, 28.647, 28.679, 28.702, 28.702, 28.724, 28.747, 28.788, 28.815, 28.838, 28.851, 28.878, 28.896, 28.924, 28.942, 28.969, 29.01, 29.041, 29.046, 29.064, 29.082, 29.118, 29.141, 29.173, 29.204, 29.231, 29.272, 29.308, 29.331, 29.354, 29.422, 29.458, 29.485, 29.485, 29.53, 29.585, 29.625, 29.662, 29.689, 29.743, 29.788, 29.824, 29.883, 29.942, 29.974, 30.051, 30.123, 30.146, 30.209, 30.295, 30.341, 30.399, 30.454, 30.494, 30.508, 30.535, 30.599, 30.671, 30.762, 30.811, 30.888, 30.943, 31.006, 31.088, 31.205, 31.341, 31.432, 31.545, 31.586, 31.69, 31.785, 31.939, 32.106, 32.183, 32.328, 32.414, 32.563, 32.731, 32.812, 34.229, 34.419, 34.687, 34.841, 35.058, 35.325, 35.506, 35.75, 35.954, 36.149, 36.33, 36.629, 36.968, 37.145, 37.394, 37.588, 37.851, 38.019, 38.421, 38.693, 38.947, 39.173, 39.494, 39.82, 39.983, 40.314, 40.789, 41.106, 41.486, 41.876, 42.238, 42.518, 42.89, 43.265, 43.768, 44.153, 44.438, 44.701, 44.95, 45.235, 45.484, 45.746, 46.068, 46.439, 46.679, 46.855, 47.123, 47.358.

Table 3 describes some important descriptive analysis of the data.

**Table 3:** Descriptive analysis.

n	Mean	Skewness	Kurtosis	Median	1^st^ Quartile	3^rd^ Quartile
294	22.051	-0.332	1.900	27.709	6.782	30.484

**Fig 3:**
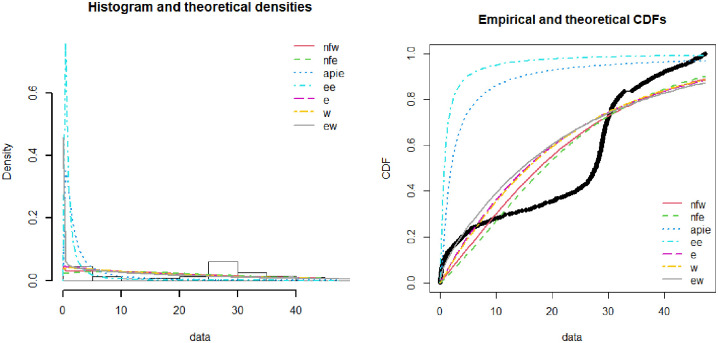
Theoretical and empirical PDF and CDF of NFW.

Fig 3 demonstrates the theoretical and empirical plots of the NFW distribution. The data is taken as the total number of death rates in millions in Pakistan during the covid-19 period. Various existing lifetime distributions like exponentiated exponential, exponential, Weibull, exponential Weibull, alpha power inverted exponential, and new flexible exponential distribution were compared with the proposed distribution. Theoretical and experimental graphs demonstrate that the proposed distribution fits the data more precisely than the existing distribution. The accuracy of both graphs can be demonstrated by the model selection criteria provided in Tables 4 and 5.

**Table 4:** MLE and standard errors for Covid-19 death rates data of Pakistan.

Model	W	A	MLE	Standard error	-log(L)
NF_Weibull	4.352	22.637	0.087	0.015	1190.364
			0.902	0.051	
NF_expo	4.202	21.814	0.062	0.002	1191.841
Ex-W	4.711	24.596	3.834	NaN	1204.855
			0.999	NaN	
			-3.796	NaN	
W	4.6793	24.424	0.042	0.008	1203.33
			1.020	0.054	
E	4.705	24.563	0.045	0.002	1203.454
APIE	NaN	NaN	8.112	0.905	1729.361
			0.446	0.027	
EE	9.812	49.355	0.004	0.000	1905.149
			0.008	0.001	
AIFW	2.756311	13.82258	0.01984159	0.002082701	1229.493
			0.05013188	0.002646834	
GAPW	4.339915	22.56649	0.36241454	0.07910969	1190.373
			0.08512305	0.01807806	
			0.90828066	0.05425457	

**Table 5:** Goodness of fit measures for Covid-19 data of Pakistan.

Models	AIC	CAIC	BIC	HQIC	P-values
NW_Weibull	2384.728	2384.769	2392.095	2387.678	4.996e-15
NF_expo	2385.681	2385.695	2389.365	2387.156	6.739e-14
E	2408.907	2408.921	2412.591	2410.383	5.362e-14
W	2410.659	2410.701	2418.027	2413.61	2.2e-16
Ex-W	2415.711	2415.794	2426.762	2420.136	2.2e-16
APIE	3463.063	3463.104	3470.43	3466.013	2.2e-16
EE	3814.298	3814.339	3821.665	3817.248	2.2e-16
AIFW	2462.986	2463.028	2470.354	2465.937	2.2e-16
GAPW	2386.745	2386.828	2397.796	2391.171	2.054e-14

**Fig 4:**
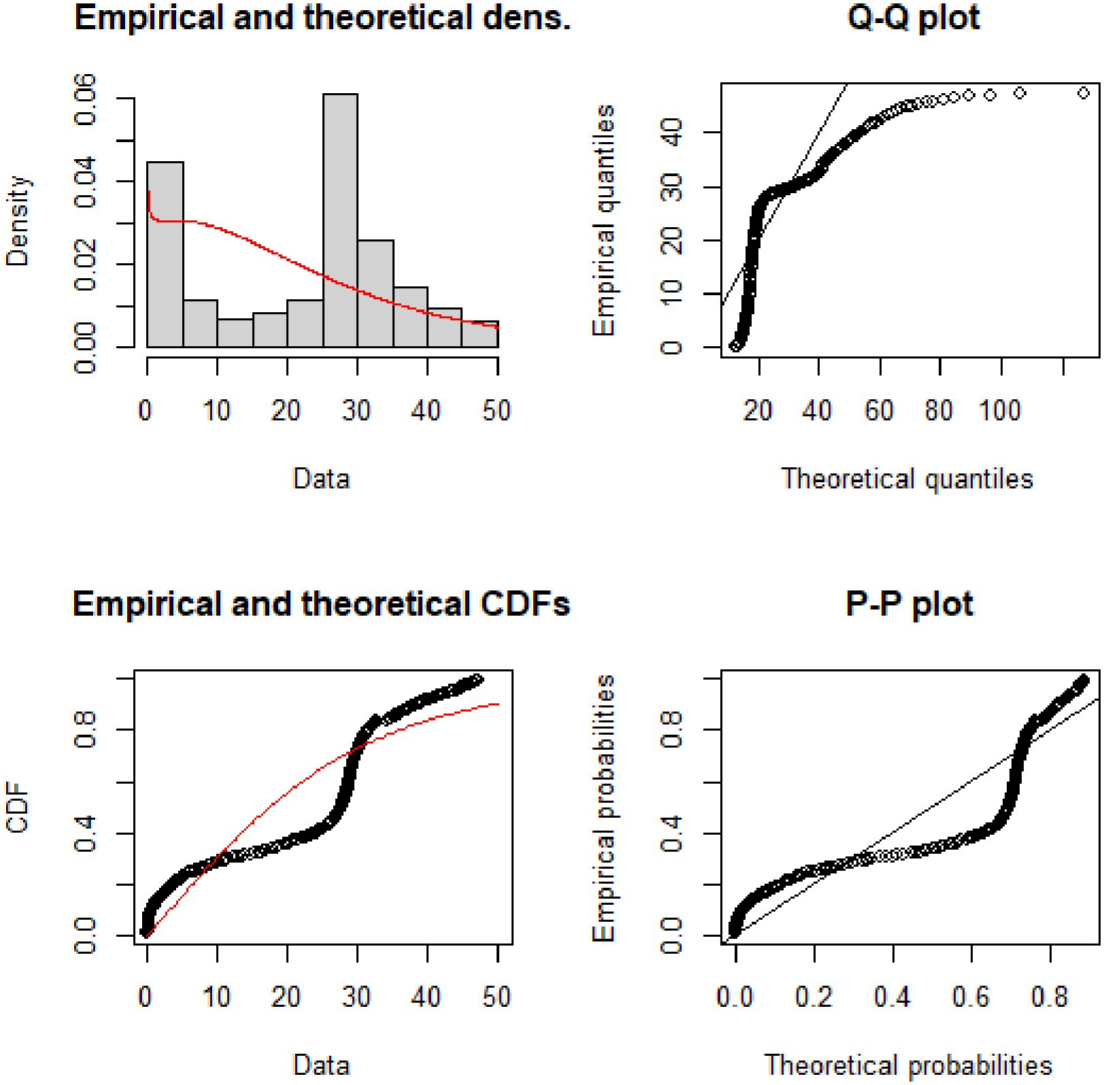
Theoretical and empirical PDF and CDF with Q-Q plot and P-P plot for NFW.

The Q-Q and P-P plots provided in Fig 4 demonstrate the covid-19 death data in millions for Pakistan. Both the Q-Q and P-P plots depict that except for the few points especially in the upper tail, the proposed NFW more reasonably describes the covid-19 death data. The NFW for the covid-19 deaths data can be described with theoretical and empirical densities.

**Fig 5:**
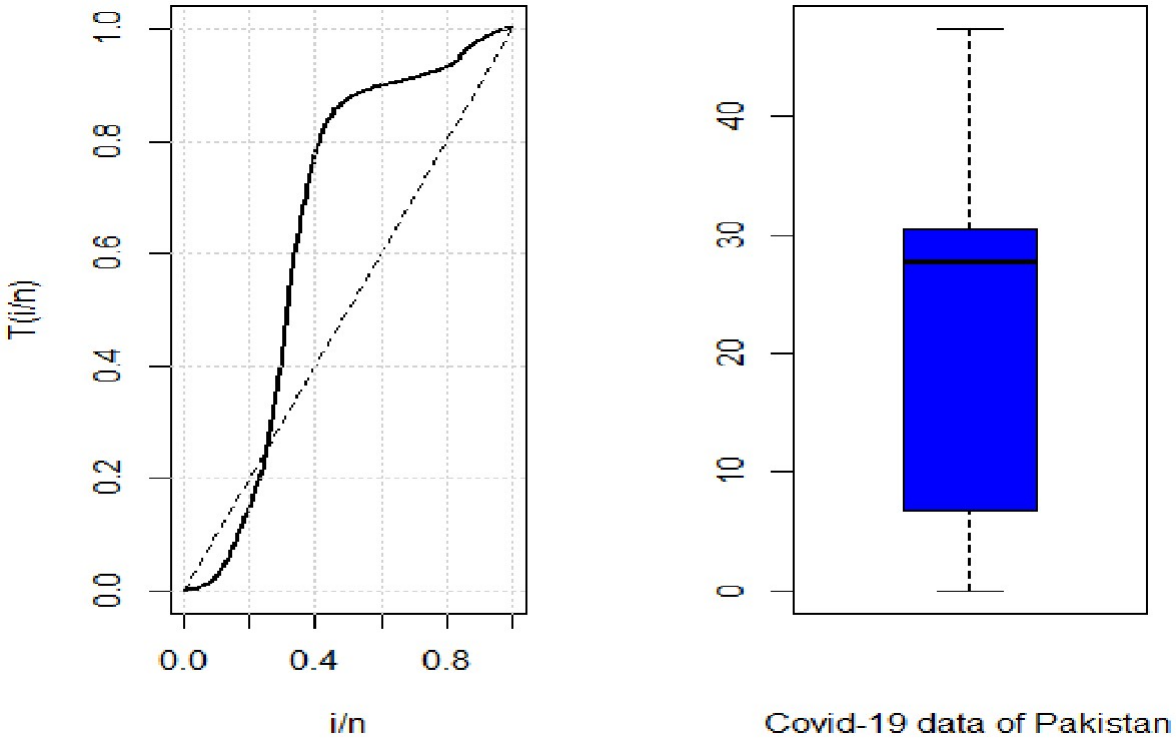
TTT and box plot of Covid-19 data for Pakistan.

Fig 5, TTT plot depicts the form of the failure rate function. The TTT plot portrays that the curve passes through the diagonal line and hence the data follows a non-monotonic failure rate function. While the box plot shows that the data is skewed to the left.

Tables 4 and 5 represent different model selection criteria, including estimates of maximum likelihood, standard error, log likelihood, Anderson-Darling (A), Cramervon-Mises (W), AIC, CAIC, BIC, HQIC, and P-values. The results in Tables 4 and 5 clearly show that, based on these model selection criteria, NFW provides a better fit when compared with exponentiated exponential, exponential, Weibull, exponential Weibull, alpha power inverted exponential, and new flexible exponential distribution.

### Data set 2: Covid-19 data for Afghanistan (Total death rates in millions)

0.026, 0.026, 0.026, 0.051, 0.077, 0.077, 0.103, 0.103, 0.103, 0.103, 0.103, 0.103, 0.206, 0.257, 0.308, 0.385, 0.411, 0.411, 0.437, 0.462, 0.462, 0.488, 0.565, 0.591, 0.745, 0.771, 0.771, 0.771, 0.848, 0.925, 0.925, 1.028, 1.028, 1.105, 1.207, 1.336, 1.49, 1.516, 1.567, 1.644, 1.747, 1.85, 2.183, 2.312, 2.44, 2.672, 2.723, 2.8, 2.954, 3.083, 3.134, 3.262, 3.391, 3.494, 3.93, 4.316, 4.367, 4.444, 4.573, 4.829, 4.984, 5.292, 5.574, 5.626, 5.651, 5.677, 5.857, 6.062, 6.345, 6.422, 6.628, 6.833, 7.039, 7.655, 7.809, 8.04, 8.503, 9.273, 9.582, 9.967, 10.506, 11.046, 11.56, 11.688, 12.202, 12.382, 12.716, 13.05, 14.129, 14.18, 14.719, 15.028, 15.336, 15.85, 16.389, 17.314, 17.519, 18.393, 18.701, 19.009, 19.318, 20.037, 20.782, 21.09, 21.27, 22.246, 23.119, 23.685, 24.121, 24.635, 24.995, 25.585, 25.996, 26.716, 27.332, 28.154, 28.694, 29.516, 29.952, 30.389, 30.441, 30.518, 30.62, 31.16, 31.519, 32.085, 32.393, 32.65, 32.675, 32.701, 32.958, 32.984, 33.009, 33.035, 33.138, 33.138, 33.292, 33.42, 33.652, 33.78, 33.934, 34.14, 34.576, 34.833, 35.064, 35.193, 35.219, 35.347, 35.398, 35.501, 35.553, 35.604, 35.604, 35.604, 35.655, 35.707, 35.912, 36.015, 36.015, 36.041, 36.041, 36.041, 36.041, 36.143, 36.22, 36.22, 36.22, 36.22, 36.297, 36.375, 36.452, 36.503, 36.503, 36.503, 36.503, 36.503, 36.657, 36.683, 36.94, 36.94, 36.965, 36.965, 37.068, 37.145, 37.171, 37.197, 37.325, 37.325, 37.376, 37.376, 37.453, 37.505, 37.505, 37.505, 37.505, 37.608, 37.608, 37.71, 37.736, 37.787, 37.813, 37.864, 37.89, 37.993, 38.044, 38.07, 38.096, 38.096, 38.198, 38.275, 38.378, 38.507, 38.558, 38.609, 38.712, 38.764, 38.866, 38.943, 39.046, 39.175, 39.329, 39.406, 39.431, 39.508, 39.508, 39.663, 39.74, 39.842, 39.997, 39.997, 40.048, 40.202, 40.51, 40.587, 40.69, 40.947, 41.05, 41.307, 41.615, 42, 42.154, 42.334, 42.463, 42.797, 43.105, 43.413, 43.721, 44.055, 44.389, 44.62, 44.698, 45.006, 45.571, 46.11, 46.804, 47.292, 47.42, 47.42, 47.883, 48.14, 48.808, 48.962, 49.296, 49.707, 49.964, 50.246, 50.477, 50.58, 51.248, 51.659, 52.019, 52.147, 52.584, 53.098, 53.483, 53.843, 54.382, 54.613, 54.947, 55.204, 55.487, 55.846, 55.975, 56.026, 56.283, 56.283, 56.283, 56.283, 57.465, 57.644.

Table 6 defines the descriptive analysis of the data.

**Table 6:** Descriptive analysis.

n	Mean	Skewness	Kurtosis	Median	1^st^ Quartile	3^rd^ Quartile
290	27.316	-0.3025102	1.754945	35.129	7.193	38.924

**Fig 6:**
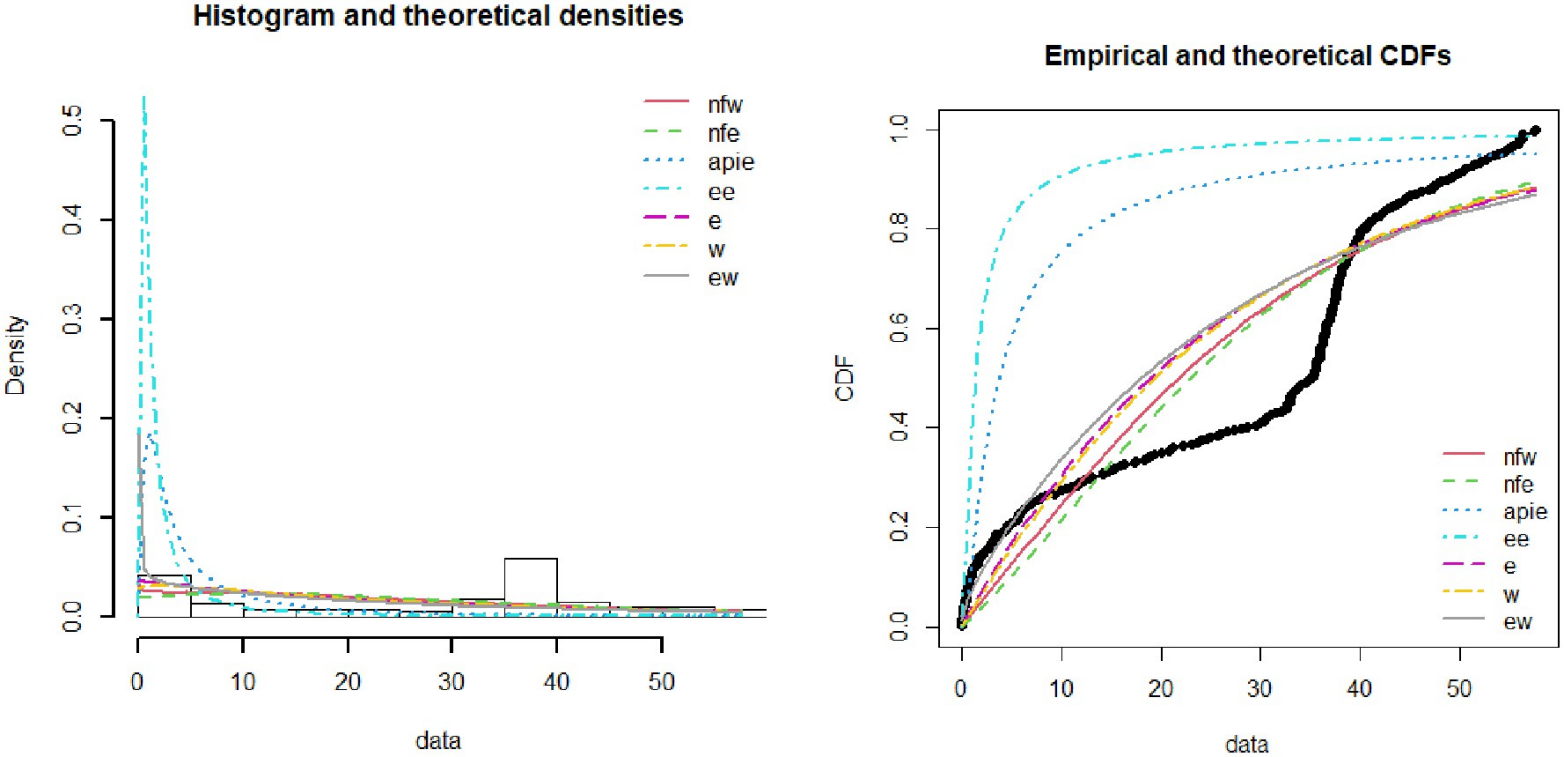
Theoretical and empirical PDF and CDF of NFW.

Fig 6 demonstrates the theoretical and empirical plots of the NFW distribution. The data were considered as the total death rate in millions for Afghanistan during covid-19. Various existing lifetime distributions like exponentiated exponential, exponential, Weibull, exponential Weibull, alpha power inverted exponential, and new flexible exponential distribution were compared with the proposed distribution. The theoretical and empirical graphs clearly demonstrate that the proposed distribution fits the data more precisely as compared with the existing distribution. The preciseness of both the graphs can be justified with the model selection criteria provided in Tables 7 and 8.

**Table 7:** MLE and standard errors for Covid-19 data of Afghanistan.

Model	W	A	MLE	Standard error	-log(L)
NF_Weibull	3.827	20.231	0.071	0.0137	1238.948
			0.905	0.0514	
NF_expo	3.698	19.522	0.050	0.0023	1240.446
Ex-W	4.063	21.519	3.137	NaN	1254.047
			0.999	NaN	
			-3.095	NaN	
W	4.0720	21.563	0.0317	0.007	1248.961
			1.0432	0.060	
E	4.122	21.839	0.0366	0.002	1249.168
APIE	7.736	39.496	31.383	4.667	1596.23
			0.7826	0.050	
EE	8.265	42.369	0.0132	0.003	1811.216
			0.0130	0.003	
AIFW	2.487	12.846	0.0457	0.004	1278.144
			0.0422	0.002	
GAPW	3.850	20.354	0.418	0.100	1238.872
			0.068	0.018	
			0.909	0.065	

**Table 8:** Goodness of fit measures for Covid-19 data of Afghanistan.

Models	AIC	CAIC	BIC	HQIC	P-values
NFWeibull	2481.896	2481.938	2489.236	2484.837	3.186e-14
NF_expo	2482.892	2482.906	2486.562	2484.362	2.417e-13
E	2500.336	2500.35	2504.006	2501.806	2.2e-16
W	2501.923	2501.965	2509.263	2504.863	2.2e-16
Ex-W	2415.711	2415.794	2426.762	2420.136	2.2e-16
APIE	3196.469	3196.51	3203.808	3199.409	2.2e-16
EE	3626.431	3626.473	3633.771	3629.372	2.2e-16
AIFW	2560.288	2560.33	2567.628	2563.228	2.2e-16
GAPW	2483.744	2483.828	2494.754	2488.155	5.884e-15

**Fig 7:**
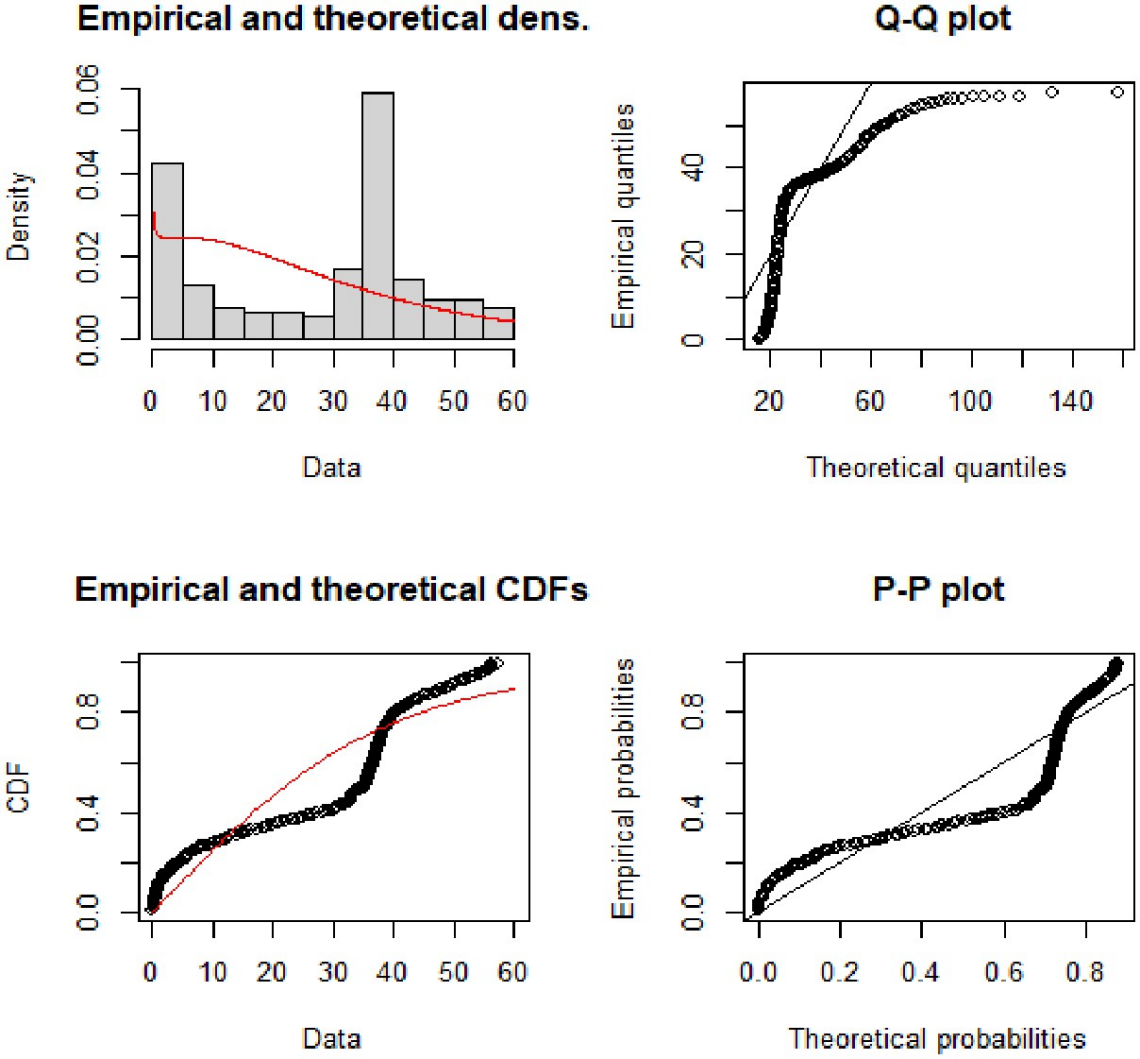
Theoretical and empirical PDF and CDF with Q-Q plot and P-P plot for NFW.

The Q-Q and P-P plots provided in Fig 7 demonstrate the covid-19 death data in millions for Afghanistan. Both the Q-Q and P-P plots depict that except for the few points especially in the upper tail, the proposed NFW more reasonably describes the covid-19 deaths data. The NFW for the covid-19 death data can be described with theoretical and empirical densities.

**Fig 8:**
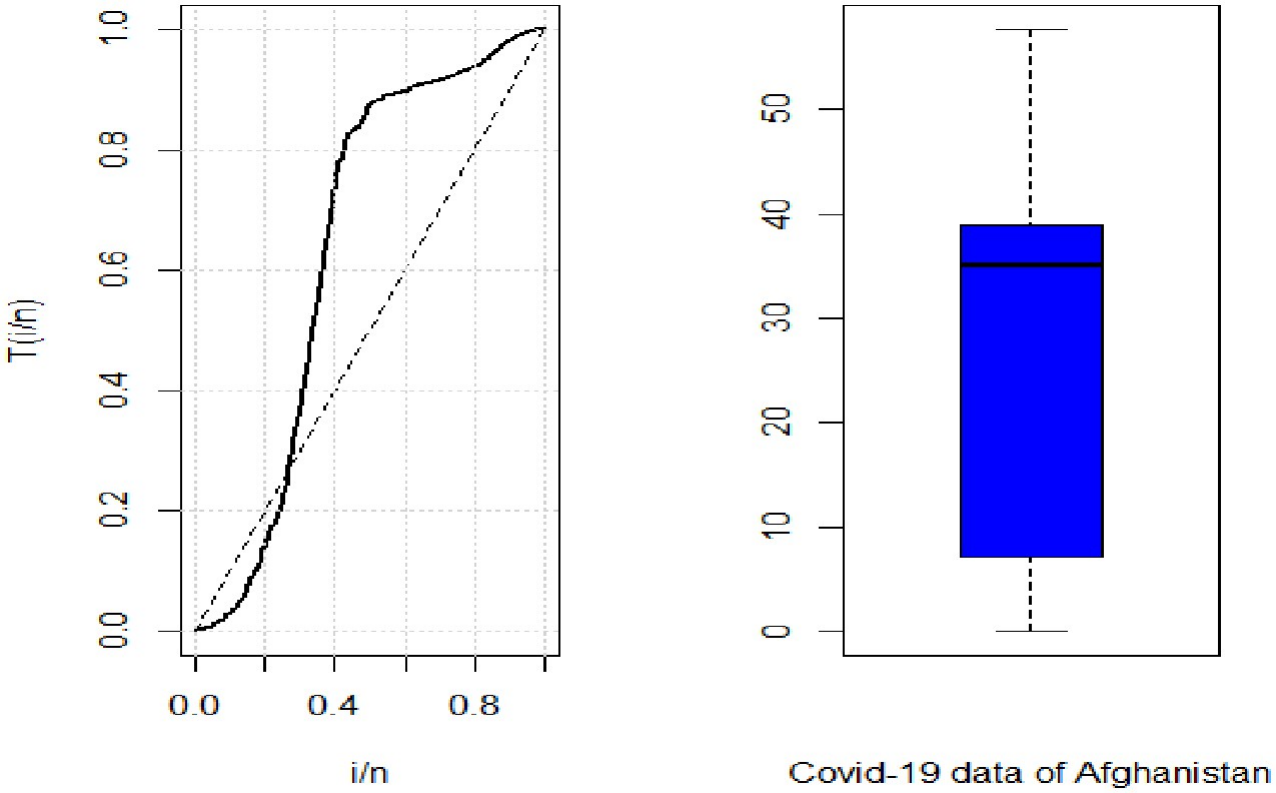
TTT and box plot of Covid-19 data for Afghanistan.

In Fig 8, TTT follows the same pattern as in Fig 5 which means that the death rate in Afghanistan also follows a non-monotonic shape. While the box plot shows that the data is left skewed.

Tables 7 and 8 represent the various model selection criteria, including maximum likelihood estimates, standard errors, log-likelihood, Anderson-Darling (A), Cramervon-Mises (W), AIC, CAIC, BIC, HQIC, and P-values. The results in Tables 7 and 8 clearly show that, based on these model selection criteria, the NFW provides a better fit as compared with exponentiated exponential, exponential, Weibull, exponential Weibull, alpha power inverted exponential, and new flexible exponential distribution.

### Monte Carlo (MC) simulation of the NFW

The simulation study is generally used when the real data is expensive or difficult to obtain. This section develops a Monte Carlo simulation (MC) study to verify the consistency of the NFW parameters. Two sets of parameters with values a = 0.29, b = 0.1 and a = 0.53, b = 0.4 are considered respectively. Bias and MSE were calculated with 1000 replicates of sample sizes n = 100, 200, 300, and 400 for both sets. The general formula for calculating bias and mean square error is given by

$$MSE=\;\frac{1}{W}{\sum_{i=1}^{W}\left(\hat{\alpha_{i}}-\alpha \right)}^{2}$$

$$Bias=\;\frac{1}{W}\sum_{i=1}^{W}\left(\hat{\alpha_{i}}-\alpha \right)$$

The general approach is that both the bias and MSE decrease as the sample size increases.

The results in Table 9 show that the MSE tends to be zero as the sample size increases, while the bias also decreases as the sample size increases.

**Table 9:** Average values of MSE and bias.

parameters	n	MSE (a)	MSE(b)	bias(a)	bias(b)
a = 0.29	50	0.04864456	0.004770595	0.2192732	0.06578161
b = 0.1	100	0.04666144	0.003921202	0.2152562	0.06097263
	150	0.04643463	0.003751044	0.2149344	0.06005994
	200	0.04585855	0.003586886	0.213689	0.05894422
a = 0.53	50	0.1180521	0.07392276	0.3405062	0.2575688
b = 0.4	100	0.1139611	0.06253756	0.3361397	0.2438181
	150	0.1124015	0.05858509	0.3341622	0.2378924
	200	0.1116605	0.0572783	0.3333947	0.2361578

## Conclusion

The government needs some predicted scores of the mortality rate of Covid-19 patients so as to plan better, and this can be done by using probability models. In this paper, best fitted model is pointed out called the NFW distribution which can leads to better estimates among others. The best fitted model captured varities of the hazard rate functions that is increasing, decreasing, J-shaped, and inverted J-shaped hazard rates. The implications of the lifetime distributions are discussed by the mortality rates of two countries. It has been stated that the NFW distribution is more suitable for modelling the mortality rate. This distribution can also be used for modeling the mortality rates of other diseases. In medical and engineering sciences, the estimation of the parameters under the bayesian paradigm plays a key role, and hence, resechers are encouraged to study the parameter estimation of this model under Bayesian paradigm.
